# Electrophysiological Quality Control of Human Dopaminergic Neurons: Are We Doing Enough?

**DOI:** 10.3389/fncel.2021.715273

**Published:** 2021-08-13

**Authors:** Vincent Seutin

**Affiliations:** Laboratory of Neurophysiology, GIGA Neurosciences, Liège University, Liège, Belgium

**Keywords:** dopaminergic neurons, Parkinson's disease, cell transplantation, excitability, human induced pluripotent stem cells

## Introduction

The breakthrough of induced pluripotent stem (iPS) cell technology (Takahashi and Yamanaka, 2006) has allowed scientists to design protocols allowing the differentiation of these cells into the main cell types of our organism. This possibility is particularly exciting in the field of Parkinson's disease (PD), since early experiments using heterologous dopaminergic (DA) or chromaffin cells have demonstrated the ability of such cells, when injected in adequate sites, to improve symptoms of the disease in a sustained manner (Kefalopoulou et al., 2014). The possibility of reproducing this effect using autologous cells, thereby free of ethical concerns, is of course fascinating (Barker et al., 2017; Parmar et al., 2020). It has clearly been demonstrated that symptoms of experimental parkinsonism can be relieved in several species by such grafts (Kikuchi et al., 2017; Song et al., 2020), with a first human case report study yielding encouraging results (Schweitzer et al., 2020). Protocols are being refined to improve the yield of DA neurons and minimize the survival of undifferentiated cells (e.g., Piao et al., 2021). Immunohistochemical methods allow to clearly define the presence of DA neurons, the absence of other monoaminergic neurons (e.g., serotoninergic neurons) and the molecular subtype of these DA neurons (by examining the expression of a variety of transcription factors such as NURR1, PITX3, etc…). Many experiments have confirmed the possibility of obtaining preparations devoid of oncogenic potential.

In the published papers, some efforts have been made to characterize the physiological properties of the DA neurons, but I argue that, in many cases, the data is not completely convincing and, more importantly, that the reader has no idea of the percentage of neurons that display the specific properties of these neurons. I believe that more standardization of these experiments is important to assess the functional quality of the future grafts. Although I reckon that safety is of paramount importance, I also believe that suboptimal functionality may impair the clinical effectiveness of future procedures.

## Functional Characterization of Dopaminergic Neurons in the Published Literature

It should be first emphasized that we do not know anything about the physiological activity of DA neurons *in situ* in the human brain (but see below). From the rodent literature, identified DA neurons from the *substantia nigra, pars compacta* (SNc) have several cardinal features: (1) they have the ability to fire action potentials spontaneously at a low frequency (0.5–5 Hz) both *in vitro* (e.g., dissociated DA neurons: Puopolo et al., 2007) and *ex vivo* in rodent brain slices (numerous studies). This slow endogenous pacemaking is very important. Indeed, ≪ in their tonic mode, DA neurons maintain a steady, baseline level of dopamine in downstream neural structures that is vital for enabling the normal functions of neural circuits ≫ (Bromberg-Martin et al., 2010). More specifically, a minimal activation of post-synaptic receptors is probably critical to ensure their appropriate sensitivity. The pacemaking activity is exquisitely regular, with the coefficient of variation (CV) of interspike intervals (ISI) usually below 0.1 (CV = standard deviation of the ISI's/Mean ISI) (de Vrind et al., 2016). (2) When examined under voltage clamp conditions, SNc DA neurons express the I_h_ current, which has a characteristic slow activation (Franz et al., 2000), as compared e.g., to hippocampal CA1 neurons. This current is a real hallmark and allows us to clearly distinguish DA neurons from GABAergic neurons in rodent slices (see also below). (3) In addition, the firing of DA neurons is normally controlled by somato-dendritic D2-type receptors. Activation of these receptors by dendritically released dopamine leads to a hyperpolarization mediated by GIRK-type K^+^ channels. This auto-inhibition may be functionally important in these neurons. (4) Some other characteristics can be observed, such as an I_A_ type K^+^ current, a medium duration after hyperpolarization mediated by SK3 channels, etc… However, these features are less characteristic of the “DA phenotype”.

In addition, *in vivo*, DA neurons can switch to burst firing and this mode induces a larger increase in synaptic and perisynaptic dopamine concentration. We and others have shown that NMDA receptor activation and down-regulation of the SK current are critical for bursting to be fully expressed (Johnson et al., 1992; Waroux et al., 2005, Zweifel et al., 2009; Soden et al., 2013). This firing pattern is critical because it usually is considered as a “GO” signal (Bromberg-Martin et al., 2010).

When recording from presumed human DA neurons a few years ago, we were struck by the similarities between the firing of some of these cells and rodent DA neurons (Borgs et al., 2016). Criteria 1–3 above were clearly satisfied in some neurons. This suggests that the physiology of *in situ* DA neurons in the human brain is similar to that of rodent cells. The more recent literature tends to confirm this assumption. Table 1 summarizes the electrophysiological characterization that was performed in recent papers describing the properties of human DA neuron preparations. In several cases, the amount of information is limited and, perhaps more importantly, the reader has no idea on whether the physiological properties were reproducible from batch to batch. One can clearly see that the information provided is limited (the second column starting from the left actually simply shows that the cells are excitable). In several cases, some of the physiological hallmarks of DA neurons have not been demonstrated to be present. Note that, in several of these studies, additional experiments showed that DA release can be measured from the cell preparations. However, given the harsh protocols used to evoke such release, this type of experiment does not guarantee that DA would be released physiologically. One other information that is usually missing is the temperature at which the experiments are carried out. This is a significant problem because pacemaking of these neurons is very temperature-dependent (Guatteo et al., 2005). Finally, a critical piece of information which is also missing is whether one or more than one batch of derived DA neurons was tested.

**Table 1:** Summary of electrophysiological hallmarks of nigral dopaminergic neurons demonstrated to be present in engineered iPS cell-derived human DA cells.

**Study**	**Ability to fire APs upon I injection**	**Spontaneous slow(<5 Hz) pacemaking**	**Presence of I_**h**_ (CC or VC)**	**Hyperpolarization by D2 agonist**	**Other**	**Remark**
Borgs et al. (2016)	Yes (11 cells)	Yes (3 cells)	Yes (3 cells)	Yes (3 cells)	–	Some properties were time-dependent
Kikuchi et al. (2017)	Yes (4 cells)	No	No	Not tested		
Song et al. (2020)	Yes (7 cells)	Yes (4 cells)	No (seems actually absent: suppl Fig. 10A)	Not tested	Population activity reported	
Kim et al. (2021)	Yes (?)	Yes° (8, 10 and 14 cells at 40, 60 and 75 DIV)	Yes (?)	Not tested	Developmental aspect of action potential duration and input resistance shown	Some properties were time-dependent
					M current suggested	
Doi et al. (2020)	Yes (16 out of 18 cells)	Yes^*^ (12 out of 18 cells in two experiments)	No	Not tested		

*? Means that the number of cells tested was not reported*.

*° The spontaneous firing shown in their figure 3M is atypical in the sense that it is quite irregular*.

* *The firing rate of the cell which was shown was 7 Hz*.

## A Proposal for Future Guidelines

From the data reviewed above, I propose the following standardized guidelines to assess the cell physiology of human DA cell preparations in future studies:

All the criteria below should be satisfied in at least 3 human iPS cell-derived DA neuron preparations made during different differentiations.

Temperature of the experiments should be close to physiological (32–35°C). The composition of the extracellular solution could be as follows: 140 mM NaCl, 5 mM KCl, 2 mM CaCl_2_, 2 mM MgCl_2_, 15 mM HEPES and 10 mM D-glucose; pH 7.4. The intrapipette solution could be as follows: 140 mM KGluconate or Kmethylsulphate, 10 mM NaCl, 0.5 mM CaCl_2_, 15 mM HEPES, 2 mM ATP-Mg, 5 mM EGTA; pH 7.4. Kmethylsulphate is preferred in general because gluconate tends to inhibit the SK-mediated conductance. Small variations around these values are of course possible, but I recommend to use a HEPES-based extracellular solution, which is appropriate for isolated cells.

- Slow spontaneous pacemaking between 0.5 and 4 Hz with a CV < 0.2 (*N* = 6 cells).
- Robust I_h_ current : as measured in voltage clamp using a pulse protocol from −60 to −120 mV, current amplitude should be ≥ 50 pA (*N* = 6 cells)
- Complete and reversible inhibition of firing (or outward current > 30 pA in voltage clamp at −60 mV) by 100 μM dopamine (*N* = 6 cells)

Criteria 1, 2 and 3 may be assessed in the same cells or in different cells. The number of experiments proposed is not based on any power calculation, but on common sense.

## Discussion

The perspective of improving the lives of parkinsonian patients in the future with autologous grafts of “their” DA neurons is very uplifting. However, in order to make it a reality, we as a community need to ensure that all aspects of the grafting procedure are optimal. I strongly believe that the physiological aspect described here is important if we are to guarantee effective grafts to the patients. We are still in a very preliminary phase in this field and it is time to standardize as much as possible the preparations. I believe that fulfilling the above criteria ensures one aspect of the standardization. One may argue that some of these criteria are not essential. For example, we cannot be sure to which extent the autoinhibition by D2 somatodendritic autoreceptors would be critical to the function of DA neurons once they are grafted. However, work in rodents demonstrates the need for DA neurotransmission to be regulated by D2 autoreceptors, which are “short” D2 (D2S) receptors. Thus specific deletion of D2S receptors disrupts the classical behavioral effect of low dose D2 agonists, as well as the regulation of tyrosine hydroxylase (TH) phosphorylation (Radl et al., 2018). Therefore, important aspects of both the biochemistry and functionality of DA neurons are dependent upon correct autoreceptor function. In addition, slow I_h_ currents of the type expressed in DA neurons are thought to be important in synaptic integration. We and others have demonstrated that, when present, the current strongly decreases temporal summation of excitatory post-synaptic potentials (EPSCs) in DA neurons (Masi et al., 2013; Engel and Seutin, 2015). When thinking about physiology, we should try to define what exactly would be the therapeutic goals of DA grafts in PD patients. Currently, about 10^6^ DA neurons are injected in three tracks in both putamens (left and right) in humans (Schweitzer et al., 2020). What do we hope from these cells? A large fraction of them will probably degenerate and an additional pool will stop expressing TH: indeed TH expression was observed in about 30% of surviving cells in a monkey study (Kikuchi et al., 2017). Experimental observations and calculations suggest that around 10^5^ cells per track would be largely enough to support recovery in humans (see a discussion about this point in Kikuchi et al., 2017). There is evidence, both from older studies using heterologous neurons in human patients and from recent preclinical studies using iPS-derived DA neurons, that the DA neurons within the graft extend their dendrites in the host tissue, become innervated and innervate targets (Kriks et al., 2011; Grealish et al., 2015). Actually, it was even shown that the Lewy body pathology can spread from the host tissue to grafted cells (Li et al., 2008). Ideally, grafted DA neurons should release low amounts of dopamine in their striatal microenvironment in a regular manner, mimicking their physiological activity and providing their “enabling function” to the basal ganglia circuitry. Possibly, if their progressive innervation is close to physiological (see Watabe-Uchida et al., 2012), one may envision that synaptic activation by some of their normal glutamatergic inputs would be able to induce bursting and to underlie the “GO” signal. However, this switch should occur appropriately, in order to prevent graft-induced dyskinesia's (see Parmar et al., 2020). Evidently, at the present time, we have no means to evaluate to what extent this would occur *in situ*. Interestingly, however, depending on the initial clinical results of the future grafts, tuning of the excitability of DA neurons could provide a means to minimize this type of problem, further emphasizing the interest of carefully evaluating this parameter.

The experiments suggested in these guidelines should of course be complemented with other experiments, in particular those aimed at determining the subtypes of SNc DA neurons that are mostly generated (Poulin et al., 2020). This will be important, because some subpopulations of SNc DA neurons, e.g., those expressing acetaldehyde dehydrogenase 1A1, appear to be more prone to neurodegeneration than others, both in rodents and humans (Poulin et al., 2020). Therefore, ensuring a high percentage of “resistant” DA neurons might be critical as well to the success of transplantation. This will also necessitate further investigations on the biology of human iPS-derived DA neurons.

## Author Contributions

The author confirms being the sole contributor of this work and has approved it for publication.

## Conflict of Interest

The author declares that the research was conducted in the absence of any commercial or financial relationships that could be construed as a potential conflict of interest.

## Publisher's Note

All claims expressed in this article are solely those of the authors and do not necessarily represent those of their affiliated organizations, or those of the publisher, the editors and the reviewers. Any product that may be evaluated in this article, or claim that may be made by its manufacturer, is not guaranteed or endorsed by the publisher.
